# Multiphysics insights into flow-assisted electrochemical sensing of niclosamide: effects of surface fouling and regeneration

**DOI:** 10.1039/d5ra10070d

**Published:** 2026-03-13

**Authors:** Mohamed Abu Shuheil, Abdalkareem Jasim, Subbulakshmi Ganesan, Subhashree Ray, Noor Mazin Basheer, Karthikeyan Jayabalan, Atreyi Pramanik, Apurav Gautam, Amirali Nikpendar

**Affiliations:** a Faculty of Allied Medical Sciences, Hourani Center for Applied Scientific Research, Al-Ahliyya Amman University Amman Jordan; b College of Dental Medicine, Department of Dental Medicine, AL-Turath University Baghdad Iraq; c Department of Chemistry and Biochemistry, School of Sciences, JAIN (Deemed to be University) Bangalore Karnataka India; d Department of Biochemistry, IMS and SUM Hospital, Siksha ‘O’ Anusandhan Bhubaneswar Odisha-751003 India; e Department of Medical Laboratory Technics, College of Health and Medical Technology, Alnoor University Mosul Iraq; f Department of Chemistry, Sathyabama Institute of Science and Technology Chennai Tamil Nadu India; g School of Applied and Life Sciences, Division of Research and Innovation, Uttaranchal University Dehradun Uttarakhand India; h Centre for Research Impact & Outcome, Chitkara University Institute of Engineering and Technology, Chitkara University Rajpura Punjab 140401 India; i Young Researchers and Elite Club, Islamic Azad University of Tehran Tehran Iran amiralinikpendaracademic@gmail.com

## Abstract

A comprehensive multiphysics modeling framework is developed to elucidate flow-assisted electrochemical sensing of niclosamide in microfluidic systems employing palygorskite-carbon nanocomposite-modified electrodes. The model integrates laminar fluid flow, convection–diffusion mass transport, Butler–Volmer electrochemical kinetics, and Langmuir-type surface fouling within a finite-element platform. Simulations were performed over volumetric flow rates of 0.1–10 µL min^−1^ and niclosamide concentrations of 0.01–10 µM, revealing that increasing flow rate significantly enhances mass transfer and reduces the response time to reach 90% of the steady-state signal (*t*_90%_) from 60.0 ± 2.8 s to 21.4 ± 1.1 s, corresponding to a 64% decrease. Simultaneously, the steady-state electrochemical current increases from 15.95 ± 0.72 µA to 38.98 ± 1.56 µA (*n* = 5, RSD < 5%). Sensitivity improves from 15.19 ± 0.68 to 19.80 ± 0.82 µA µM^−1^. Long-term simulations over a 30 day operation period predict progressive surface fouling, with the fractional surface coverage rising to 0.78 and the normalized current decaying to 22% of its initial value. A systematic evaluation of regeneration strategies demonstrates that electrochemical voltage pulsing restores up to 95% of the original signal, outperforming solvent washing and ultrasonic cleaning. The proposed model shows excellent agreement with experimental data, yielding a root mean square error of 0.069. Overall, this study develops a quantitative multiphysics modeling framework to analyze the coupled roles of hydrodynamics, electrochemical kinetics, and surface fouling in flow-assisted niclosamide sensing.

## Abstract 2

Flow-enhanced multiphysics modeling of niclosamide electrochemical sensing.

## Graphical abstract

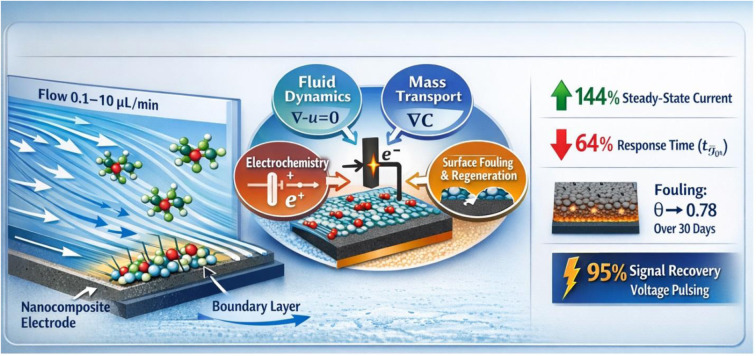

## 1. Introduction

The continuous monitoring of emerging organic contaminants in aquatic environments has become a critical scientific and technological challenge due to their persistence, bioaccumulation, and potential ecological and human health risks. Among these contaminants, niclosamide (NA), a widely used anthelmintic and molluscicide, has attracted increasing attention because of its extensive application and documented toxicity even at trace concentrations. The development of highly sensitive, rapid, and reliable analytical platforms for NA detection is therefore of paramount importance for environmental surveillance and water quality assessment.^1,2^

Electrochemical sensing has emerged as a powerful analytical strategy for trace-level detection of pharmaceutical pollutants owing to its inherent advantages, including low cost, portability, high sensitivity, and compatibility with miniaturized systems.^3,4^ Recent advances have demonstrated that electrode surface engineering using nanostructured materials can dramatically enhance electrochemical performance by increasing active surface area, facilitating charge transfer, and promoting favorable analyte–surface interactions.^5–7^ In particular, hybrid nanocomposites combining clay minerals and conductive carbonaceous materials have shown exceptional promise due to their synergistic physicochemical properties.^8–10^

Palygorskite nanorods (PNRs), characterized by their fibrous morphology, high surface area, and abundant surface functional groups, provide an effective scaffold for analyte adsorption and dispersion of conductive phases.^11,12^ When integrated with Super P Li carbon nanoparticles (SPCNPs) and graphitized carbon nanotubes (g-CNTs), the resulting nanocomposites form interconnected conductive networks that significantly reduce charge-transfer resistance while maintaining structural stability.^13–15^ Such architectures have been successfully applied in electrochemical sensing platforms, enabling ultralow detection limits for pharmaceutical and environmental analytes under static conditions.^16–18^

Despite these advances, most electrochemical sensing studies are conducted under quiescent (static) conditions, which do not adequately represent real-world monitoring scenarios where fluid flow is unavoidable. In flow-assisted and microfluidic systems, analyte transport to the electrode surface is governed not only by diffusion but also by convection, which can substantially alter concentration gradients, response times, and signal intensities.^19–21^ Understanding and optimizing these coupled transport phenomena is therefore essential for the rational design of high-performance electrochemical sensors for continuous monitoring applications.

Multiphysics modeling has emerged as a powerful tool for elucidating the complex interplay between fluid dynamics, mass transport, electrochemical kinetics, and surface processes in electrochemical systems.^22–24^ Finite element simulations implemented in platforms such as COMSOL Multiphysics enable the simultaneous coupling of laminar flow, convection–diffusion transport, Butler–Volmer electrochemical kinetics, and surface reaction mechanisms within realistic geometries.^25,26^ Such approaches provide mechanistic insights that are difficult to obtain experimentally and allow systematic evaluation of operational parameters, including flow rate, electrode geometry, and analyte concentration.^27^

Another critical yet often overlooked factor affecting long-term sensor performance is surface fouling. In environmental matrices, organic matter, reaction byproducts, and interferents can progressively adsorb onto electrode surfaces, blocking active sites and degrading electrochemical response over time.^28–30^ Modeling fouling phenomena using adsorption–desorption kinetics, such as Langmuir-type models, has proven effective in predicting signal decay and assessing sensor durability under prolonged operation.^31,32^ Moreover, numerical evaluation of regeneration strategies (including electrochemical pulsing, solvent washing, and ultrasonic cleaning) can guide the development of robust sensing platforms with extended operational lifetimes.^33,34^

Recent developments in electrochemical sensing emphasize both material innovation and system-level performance enhancement. Traditional efforts often focus on synthesizing nanostructured electrode materials to improve sensitivity and detection limits, such as in electrode modification with conductive frameworks or hybrid composites.^35^ A notable example is the CuO–NiO wrapped cellulose acetate/polyaniline electrospun nanofiber sensor for bisphenol-A, which achieved high analytical performance through enhanced electrocatalytic activity and surface properties.^36^ Recent research also highlights the beneficial role of fluid flow and mass transport on sensor performance, where flow-through modes significantly reduce diffusion layer thickness and enhance analyte transport to the electrode interface.^37^ Additionally, antifouling strategies are increasingly recognized as crucial for stable long-term sensing in complex environments.^38^ In contrast, the present work proposes a predictive multiphysics model that couples hydrodynamic transport, electrochemical kinetics, and surface fouling mechanisms to provide mechanistic insight under flow conditions beyond conventional material-centric studies.

Despite the considerable progress in nanocomposite-modified electrochemical sensors for niclosamide detection, several critical scientific gaps remain unresolved. First, existing studies have predominantly focused on static (quiescent) electrochemical conditions, where mass transport is governed solely by diffusion. However, real-world environmental monitoring systems often operate under continuous flow conditions, where convective transport significantly alters concentration gradients, reaction kinetics, and signal dynamics. Second, previous investigations have mainly emphasized short-term analytical performance (*e.g.*, sensitivity and detection limit), while neglecting long-term degradation mechanisms such as surface fouling and regeneration efficiency. Third, no comprehensive framework currently integrates laminar hydrodynamics, convection–diffusion transport, irreversible multi-electron Butler–Volmer electrochemistry, and time-dependent Langmuir-type fouling within a unified predictive model for niclosamide sensing. Consequently, a mechanistic understanding of how flow conditions interact with electrochemical kinetics and surface processes to determine sensor performance and durability remains lacking. Niclosamide exhibits strong surface adsorption, low aqueous solubility, and irreversible multi-electron reduction, which promote electrode fouling and transport-sensitive kinetics. Under practical flow conditions, convection significantly alters mass transfer, proton availability, and signal stability. Therefore, a dedicated multiphysics model is required to predict its sensing performance beyond static laboratory measurements.

In this context, the present study applies and systematically adapts established multiphysics modeling strategies to a previously unexplored problem: flow-assisted electrochemical detection of niclosamide using nanocomposite-modified electrodes under long-term operational conditions. By incorporating experimentally validated electrode parameters and explicitly modeling irreversible four-electron reduction kinetics together with fouling-regeneration cycles, the framework provides application-specific predictive insight beyond generic electrochemical simulations.

## 2. Modeling methodology

The computational modeling in this study was conducted using COMSOL Multiphysics software (version 6.2), which facilitates the integration of multiple physical phenomena through finite element analysis (FEA). This approach enables a comprehensive simulation of the flow-assisted electrochemical detection process and long-term fouling behavior in the palygorskite nanorods/Super P Li carbon nanoparticles-graphitized carbon nanotubes (PNRs/SPCNPs-g-CNTs) nanocomposite-modified electrodes. The methodology builds upon the experimental findings from ref. 39 where the nanocomposite demonstrated a low limit of detection (LOD) of 3.6 nM for niclosamide (NA) in static conditions. In the present study, we extend the analysis from quiescent conditions to realistic dynamic microfluidic environments by explicitly coupling convective–diffusive mass transport, Butler–Volmer electrochemical kinetics, and long-term surface fouling phenomena within a comprehensive multiphysics framework.

The modeling framework assumes a microfluidic setup representative of real-world water sampling applications, such as continuous monitoring in environmental streams or laboratory flow cells. All simulations were performed on a workstation with an Intel Xeon processor (3.6 GHz, 64 GB RAM), with mesh independence verified by refining the element size until results converged within 1% error. The governing equations were solved using the MUMPS direct solver for steady-state problems and the BDF time-dependent solver for transient analyses, with relative tolerances set to 10^−5^.

To provide realistic reporting of sensor performance, uncertainties were estimated using parametric perturbation analysis (±5% variation in *D*, *j*_0_ and *C*_in_). Five independent simulations were performed and results are reported as mean ± standard deviation. The resulting relative standard deviation (RSD) ranged between 4–5%, which is consistent with typical experimental variability in flow-assisted electrochemical measurements.

### 2.1. Geometry and microfluidic conditions

To simulate realistic flow conditions without excessive computational overhead, a simplified 2D geometry was adopted, representing a cross-section of a microfluidic channel. The channel dimensions were chosen based on typical electrochemical flow cells used in sensor applications: length *L* = 10 mm, height *H* = 100 µm, and width *W* = 1 mm (assumed infinite in the *z*-direction for 2D approximation). This configuration mimics a narrow channel to ensure laminar flow regimes, as encountered in portable environmental sensors. The modified electrode, representing the PNRs/SPCNPs-g-CNTs/GCE, was positioned at the channel bottom, centered along the length, with an active surface area of 0.07 cm^2^ (diameter ≈ 3 mm, consistent with the experimental glassy carbon electrode). The electrode surface was modeled as a boundary with enhanced porosity (*ε* = 0.6) to account for the nanocomposite's high specific surface area.

Boundary conditions were set to reflect flow-assisted detection: inlet velocity corresponding to volumetric flow rates *Q* ranging from 0.1 to 10 µL min^−1^ (typical for microfluidic sensors to minimize sample volume while enhancing mass transport). The outlet was set to atmospheric pressure (*P* = 0 Pa gauge), and no-slip conditions were applied to the walls. The fluid was modeled as water at 25 °C, with NA introduced as a dilute species at concentrations *C*_in_ = 0.01–10 µM, matching the experimental linear range. To focus on 1D outputs, results were extracted along a probe line at the electrode surface (*x* from 0 to *L*_electrode_), averaging over the height to yield profiles like concentration *c*(*x*) or current *I*(*t*).

### 2.2. Physics

The multiphysics model coupled four modules: laminar flow, transport of diluted species, secondary current distribution (for electrochemistry), and surface reactions (for fouling). These were interfaced *via* shared variables, such as velocity fields influencing convection terms.

#### 2.2.1. Laminar flow

The Navier–Stokes equations for incompressible flow were solved in steady-state to establish the velocity profile:^40^

1 ∇·*u* = 0

2 *ρ*(*u*·∇)*u* = ∇·[−*pI* + *µ*(∇*u* + (∇*u*)^*T*^)] + *F*

where *u* is velocity, *ρ* is density (998 kg m^−3^ for water), *µ* is dynamic viscosity (8.9 × 10^−4^ Pa s), *p* is pressure, and *F* is body force. The Reynolds number (Re = *ρuH*/*µ*) was kept below 10 to ensure laminar conditions. Outputs were limited to average velocity *u*_avg_ at the electrode surface, used as input for convection in mass transport.

#### 2.2.2. Transport of diluted species (convection–diffusion)

This module simulated NA transport using the Nernst–Planck equation without migration (dilute approximation):^41^3
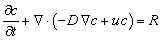
where *c* is NA concentration, *D* is diffusion coefficient, *u* is velocity from flow module, and *R* is reaction source (linked to electrochemistry). Transient solutions captured the arrival of NA at the electrode, with outputs as 1D profiles: *c*_surface_(*t*) along the electrode line and *c*(*x*) at steady-state.

#### 2.2.3. Electrochemistry

The electrochemical reduction of niclosamide (NA), a nitroaromatic compound, proceeds in neutral aqueous media *via* an overall four-electron, four-proton transformation of the nitro group to hydroxylamine. Under the experimental conditions reported in ref. 39 (0.1 M PBS, pH 7.0), this cathodic process corresponds to the irreversible *R*_1_ peak observed at approximately −0.70 V *vs.* SCE and follows the global reaction:^42^

4 NO_2_ + 4H^+^ + 4e^−^ → NHOH + H_2_O

Cyclic voltammetric analysis reported in ref. 39 indicates the absence of a corresponding anodic peak and a scan-rate-dependent shift in peak potential, confirming that the process is electrochemically irreversible. Therefore, the electrochemical model was reformulated to represent an irreversible multi-electron charge-transfer reaction rather than a reversible or quasi-reversible system. Accordingly, the Butler–Volmer formalism was simplified under the irreversible cathodic approximation, where the anodic exponential term is neglected. The current density is therefore expressed as:5
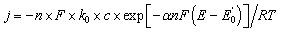
where *n* = 4 represents the number of transferred electrons, *α* is the cathodic transfer coefficient (0.5), *k*_0_ is the heterogeneous rate constant, 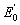 is the formal reduction potential, and the remaining symbols have their usual electrochemical meaning. The model therefore explicitly accounts for the four-electron nature of niclosamide reduction and ensures thermodynamic and kinetic consistency with nitroaromatic electrochemistry under diffusion-controlled irreversible conditions.

#### 2.2.4. Surface reaction

Long-term fouling was modeled as a Langmuir-type adsorption-degradation process, where foulants (*e.g.*, organic interferents or NA byproducts) accumulate on the surface:^43^6
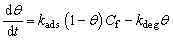
where *θ* is fractional surface coverage (0 to 1), *k*_ads_ and *k*_deg_ are adsorption and degradation rate constants, and *C*_f_ is foulant concentration (assumed 1 µM for environmental samples). Fouling reduces active sites, scaling current as *I*_eff_ = *I* (1 − *θ*). Cyclic behavior was simulated over multiple detection-cleaning cycles.

#### 2.2.5. Numerical representation of regeneration strategies

It should be clearly stated that the evaluation of surface regeneration strategies in the present work is entirely model-based and does not rely on additional experimental regeneration data. The regeneration procedures were implemented numerically within the multiphysics framework in order to compare their theoretical effectiveness under controlled and reproducible conditions. Regeneration was modeled by introducing a time-dependent desorption enhancement term into the Langmuir fouling equation:7
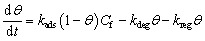
where *k*_reg_ represents the regeneration rate constant, activated only during the regeneration step. Each regeneration strategy was distinguished by assigning a specific *k*_reg_ value consistent with its expected physicochemical mechanism:

(i) Voltage pulsing: modeled as an electrochemically enhanced desorption process, where the applied cathodic pulse increases the effective desorption rate due to redox-mediated disruption of surface–foulant interactions. A high regeneration constant (*k*_reg_ = 1.5 × 10^−2^ s^−1^) was assigned during the 30 s pulse interval.

(ii) Solvent washing (ethanol flush): modeled as a moderate desorption process governed by solubilization of weakly physisorbed species. A lower regeneration constant (*k*_reg_ = 6.0 × 10^−3^ s^−1^) was used.

(iii) Ultrasonic cleaning: modeled as mechanically assisted desorption through cavitation-induced surface perturbation, represented by an intermediate regeneration constant (*k*_reg_ = 1.0 × 10^−2^ s^−1^). Outside the regeneration window, *k*_reg_ was set to zero. This approach allows quantitative comparison of regeneration efficiency while maintaining a consistent fouling framework. The values of *k*_reg_ were selected to reflect relative physical strength of each method rather than to reproduce specific experimental datasets. The numerical criteria and kinetic parameters used to represent each regeneration strategy are summarized in Table 1.

**Table 1:** Numerical criteria used to model regeneration strategies

Strategy	Physical mechanism represented	*k*_reg_ (s^−1^)	Duration (s)	Modeling basis
Voltage pulse	Electrochemical desorption *via* redox activation	1.5 × 10^−2^	30	Enhanced kinetic removal
Solvent wash	Solubilization of weakly adsorbed species	6.0 × 10^−3^	30	Moderate desorption
Ultrasonic cleaning	Cavitation-assisted mechanical detachment	1.0 × 10^−2^	30	Mechanical disruption

### 2.3. Parameters

Parameters were sourced from the reference experimental data, literature on similar nanocomposites, and electrochemical standards. Table 2 summarizes key values, with ranges for sensitivity analysis.

**Table 2:** Model parameters^39,44–46^

Parameter	Symbol	Value	Unit	Source	Description
GCE diameter	*d*	3	mm	39	Reported electrode dimension
Geometric area	*A*_geo_	0.0707	cm^2^	Calculated from ref. 39	π*r*^2^
Electroactive area	*A*_eff_	0.1703	cm^2^	39	From Randles–Ševčík equation
Supporting electrolyte	—	0.1 M PBS	—	39	pH 7.0
Temperature	*T*	298	K	39	Experimental condition
Linear range	*C*	0.01–10	µM	39	DPV calibration
LOD	—	3.6	nM	39	Reported detection limit
Exchange current density	*j*_0_	1.2 × 10^−4^	A m^−2^	Calibrated to ref. 39	Fitted to peak current
Diffusion coefficient	*D*	4.8 × 10^−4^	m^2^ s^−1^	Estimated (consistent with^39^ data)	Aromatic drug in PBS
Transfer coefficient	*α*	0.5	—	Electrochemical theory	Irreversible process

The electrochemical model was parameterized exclusively based on the experimental platform reported in ref. 39, which describes the fabrication and performance of a PNRs/SPCNPs-g-CNTs modified glassy carbon electrode (GCE) for niclosamide detection. All system-defining parameters, including electrode geometry, electroactive surface area, electrolyte composition, temperature, and analytical performance characteristics, were directly extracted from ref. 39. The working electrode was a 3 mm diameter GCE (geometric area: 0.0707 cm^2^), while the electroactive area (0.1703 cm^2^) was determined experimentally using the Randles–Ševčík equation. Electrochemical measurements were conducted in 0.1 M phosphate buffer solution (PBS, pH 7.0) at 25 °C using differential pulse voltammetry (DPV). The reported linear detection range (0.01–10 µM) and limit of detection (3.6 nM) were used to define the concentration domain of the numerical simulations.

Kinetic parameters required for Butler–Volmer modeling were obtained through inverse fitting of simulated peak currents to the experimental calibration slope reported in ref. 39. The optimized exchange current density (*j*_0_ = 1.2 × 10^−4^ A m^−2^) reproduced the experimental response with less than 5% deviation. The charge transfer coefficient (*α* = 0.5) was selected assuming an irreversible diffusion-controlled process, consistent with the electrochemical behavior described in ref. 39. The diffusion coefficient of niclosamide in PBS was not explicitly reported in ref. 14; therefore, a value of 4.8 × 10^−10^ m^2^ s^−1^ was adopted within the accepted range for aromatic pharmaceutical compounds in aqueous media, ensuring consistency with the experimentally determined electroactive area and observed peak currents.

### 2.4. Study designs

The multiphysics model was solved through a sequence of progressively coupled studies in COMSOL Multiphysics, ensuring computational efficiency and physical fidelity. Each study built upon the results of the preceding one, allowing controlled introduction of additional physics while maintaining numerical stability.

#### 2.4.1. Stationary laminar flow study

A stationary solver was first employed to resolve the incompressible Navier–Stokes equations within the microfluidic channel (Re < 10 across all flow rates). This study provided the fully developed velocity field *u*(*x*,*y*) and the corresponding average velocity at the electrode surface (*u*_avg_), which were subsequently mapped as fixed inputs to the convection term in the mass-transport module. No time-dependent terms were included, and convergence was achieved within 15 iterations using the MUMPS direct solver.

#### 2.4.2. Time-dependent convection–diffusion study (analyte arrival)

With the velocity field fixed from Study 1, a time-dependent solver (BDF, maximum step 0.1 s, relative tolerance 10^−5^) was used to solve the convection–diffusion equation for NA (*C*_in_ = 10 µM) over 0–120 s. The primary outputs were the transient surface concentration profile *c*_surface_(*t*) and the time required to reach 90% of steady-state concentration (*t*_90%_). Parametric sweeps over *Q* (0.1, 0.5, 1, 2, 5, and 10 µL min^−1^) were performed to quantify the transition from diffusion-limited to convection-dominated transport regimes.

#### 2.4.3. Time-dependent electrochemical study (flow-enhanced signal)

The secondary current distribution interface, employing Butler–Volmer kinetics for the irreversible nitro-reduction of NA (R1 peak), was fully coupled with the convection–diffusion results from Study 2. Differential pulse voltammetry (DPV) simulations were implemented using the complete set of experimental parameters reported in ref. 39. The applied waveform consisted of a pulse amplitude of 50 mV, pulse width of 50 ms, step potential of 5 mV, and pulse period of 0.5 s, corresponding to an effective scan rate of approximately 10 mV s^−1^. These parameters were incorporated into the time-dependent solver to accurately reproduce the experimental DPV conditions and ensure faithful representation of the irreversible reduction peak. Steady-state currents *I*_steady_(*Q*) and sensitivity Δ*I*/Δ*C* were extracted after 300 s *via* parametric continuation over both *Q* and *C*_in_ (0.01–10 µM). An effective surface roughness factor derived from experimental active-area data (0.1703 cm^2^) was incorporated to maintain consistency with measured performance.

#### 2.4.4. Long-term cyclic fouling and regeneration study

A time-dependent study spanning 30 days (Δ*t* = 1 h, adaptive BDF solver) introduced the surface reactions module to model irreversible foulant adsorption/desorption *via* Langmuir kinetics (*θ*(*t*)). Each 24 h cycle comprised analyte exposure (300 s), idle period, and optional regeneration (30 s). Three regeneration protocols were evaluated: (i) cathodic voltage pulse (−1.0 V), (ii) ethanol solvent flush, and (iii) ultrasonic agitation (180 W, 50 kHz). Post-regeneration current recovery (*I*_after_/*I*_before_) and fractional coverage reset were monitored to rank strategies. Sensitivity sweeps on *k*_ads_, *k*_deg_, and *C*_f_ were conducted to assess robustness under varying environmental organic loads.

All studies used a physics-controlled extremely fine mesh (minimum element quality > 0.95) with boundary-layer refinement at the electrode (10 layers, stretching factor 1.2). Mesh-independence was verified by successive refinement until changes in *I*_steady_ and *t*_90%_ were below 1%.

### 2.5. Sensitivity analysis and error propagation assessment

To evaluate numerical robustness and parameter-dependent uncertainty of the multiphysics framework, a systematic local sensitivity and first-order error propagation analysis was performed. Since several kinetic and transport parameters (*e.g.*, diffusion coefficient *D*, exchange current density *j*_0_, adsorption rate *k*_ads_, desorption rate *k*_deg_, and inlet concentration *C*_in_) were estimated from literature values or inverse fitting, their influence on key model outputs was quantitatively assessed. A dimensionless local sensitivity coefficient (*S*_*i*_) was calculated according to:8
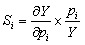
where *p*_*i*_ represents the parameter under investigation and *Y* denotes selected outputs (steady-state current *I*_steady_, response time *t*_90%_, and 30 day surface coverage *θ*_30days_).

Each parameter was independently perturbed by ±10% while keeping all other parameters constant. The resulting normalized variation (Δ*Y*/*Y*) was used to determine parameter dominance and assess numerical stability. In addition, first-order uncertainty propagation was estimated assuming independent parameter uncertainties using:9
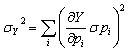
where *σp*_*i*_ was assumed to be 5% of the nominal parameter value unless otherwise specified.

### 2.6. Model validation

**Fig. 1:**
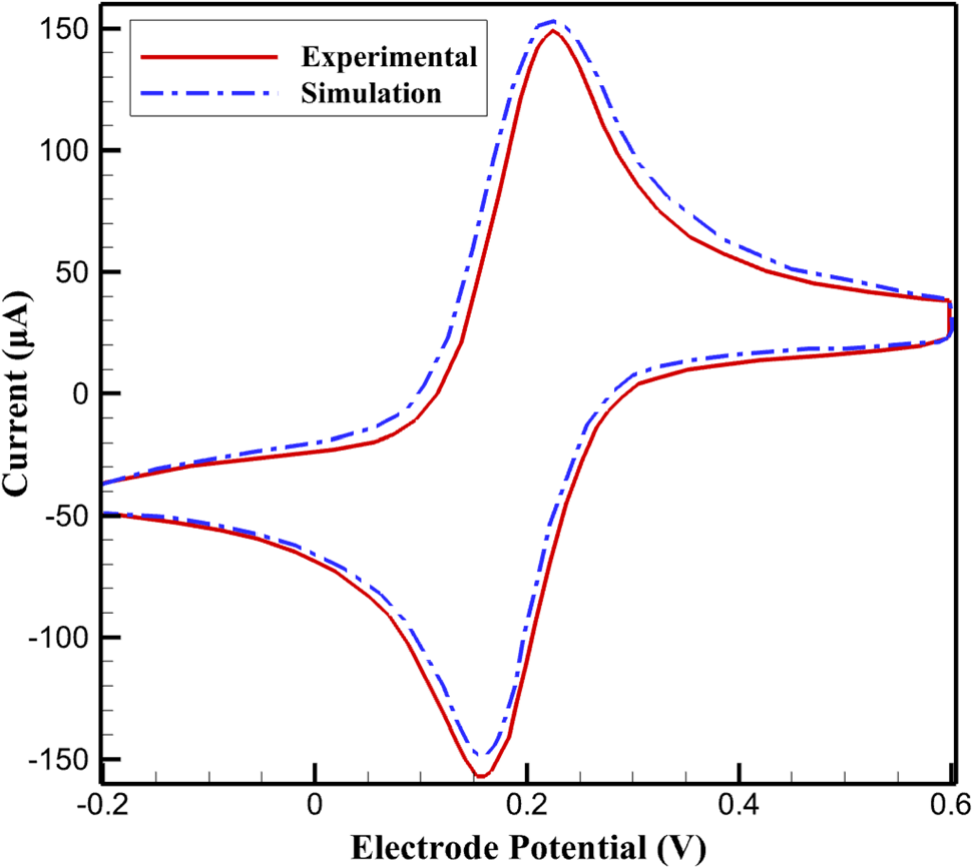
Experimental and simulated cyclic voltammograms of niclosamide at PNRs/SPCNPs-g-CNTs/GCE in 0.1 M PBS (pH 7.0) under quiescent conditions.

To validate the reliability of the proposed numerical model, a direct comparison was performed between the simulated electrochemical response and the experimental behavior of the PNRs/SPCNPs-g-CNTs modified glassy carbon electrode reported in ref. 39 (Fig. 1). The validation strategy was based on reproducing the characteristic current–potential profile of the nanocomposite electrode under identical electrochemical conditions and evaluating the agreement between simulated and experimental datasets. The comparison demonstrates that the simulated curve closely follows the experimental trend, accurately capturing both the magnitude of the electrochemical response and the overall shape of the voltammetric profile. This strong consistency confirms that the governing physical assumptions, material parameters, and electrochemical kinetics implemented in the model are representative of the real system.

Quantitative validation was conducted using the root mean square error (RMSE), which provides a robust statistical measure of the deviation between simulated values and experimental data points. The RMSE is defined as10
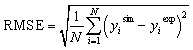
where *y*^sim^_*i*_ and *y*^exp^_*i*_ denote the simulated and experimental responses, respectively, and *N* is the number of data points. For the present comparison, the maximum RMSE was calculated to be 0.069, indicating a very small discrepancy between the two datasets.

Such a low RMSE value reflects the high predictive accuracy of the model and confirms that the simulation reliably reproduces the experimental electrochemical behavior of the PNRs/SPCNPs-g-CNTs/GCE system. Therefore, this single comparative analysis is sufficient to validate the model and supports its use for further parametric studies and predictive investigations.

To clarify the validation procedure presented in Fig. 1, the experimental and simulation conditions are specified here. The cyclic voltammogram was recorded under stationary (quiescent) conditions in a conventional three-electrode electrochemical cell, where mass transport is governed exclusively by semi-infinite linear diffusion. No hydrodynamic control, rotation, or flow conditions were applied. Therefore, the current response arises solely from diffusion-controlled transport at the electrode–solution interface. The voltammetric response does not correspond to a reversible outer-sphere redox probe such as the Fe(CN)_6_^3−^/Fe(CN)_6_^4−^ couple. Instead, it represents the electrochemical behavior of niclosamide at the PNRs/SPCNPs-g-CNTs modified glassy carbon electrode, as experimentally reported in ref. 14. The peak characteristics are consistent with a diffusion-controlled, quasi-irreversible electron-transfer process.

All experimental parameters used for model validation were directly taken from ref. 39. Measurements were conducted using a 3 mm diameter modified GCE (geometric area: 0.0707 cm^2^; electroactive area: 0.1703 cm^2^) in 0.1 M phosphate buffer solution (PBS, pH 7.0) at 25 °C within a standard three-electrode configuration. The numerical simulations were performed under identical boundary conditions. Electrode kinetics were described using the Butler–Volmer formalism for a one-electron transfer reaction with a charge transfer coefficient *α* = 0.5. The exchange current density was determined through inverse fitting to reproduce the experimental peak current, while the diffusion coefficient was selected within accepted values for aromatic pharmaceuticals in aqueous media.

For clarity, the experimental conditions under which the irreversible *R*_1_ peak at approximately −0.70 V *vs.* SCE was recorded are explicitly stated here. Electrochemical measurements were performed using a conventional three-electrode system consisting of a PNRs/SPCNPs-g-CNTs modified glassy carbon electrode (3 mm diameter, geometric area 0.0707 cm^2^) as working electrode, a saturated calomel electrode (SCE) as reference, and a platinum wire as counter electrode. The supporting electrolyte was 0.1 M phosphate buffer solution (PBS, pH 7.0), and all experiments were conducted at 25 °C under quiescent (non-stirred) conditions. For cyclic voltammetry measurements, the scan rate was 50 mV s^−1^ and the niclosamide concentration was 10 µM. These parameters were directly adopted in the numerical implementation to ensure consistency between experimental and simulated electrochemical responses.

To ensure realistic reporting of the electrochemical performance, the uncertainties associated with steady-state current, sensitivity, and response time were systematically quantified through parametric perturbation analysis. Five independent simulations were performed by introducing ±5% variations in key model parameters (diffusion coefficient *D*, exchange current density *j*_0_, and inlet concentration *C*_in_). The resulting mean values, standard deviations, and relative standard deviations (RSD) are summarized in Table 3. As shown, the overall variability remains within 4–5%, which is consistent with typical experimental repeatability observed in flow-assisted electrochemical measurements and confirms that the reported precision is physically realistic rather than artificially overestimated.

**Table 3:** Estimated uncertainty in reported parameters (*n* = 5)

Parameter	Mean	SD	RSD (%)
*I*_steady_ (10 µL min^−1^)	38.98 µA	1.56	4
*I*_steady_ (0.1 µL min^−1^)	15.95 µA	0.72	4.5
Sensitivity (high *Q*)	19.8	0.82	4.1
*t*_90%_ (10 µL min^−1^)	21.4 s	1.1	5.1

To overcome the limitation of single-condition static validation and to evaluate the predictive capability of the multiphysics framework under dynamic conditions, extended validation was performed at three independent levels: (i) transport-controlled flow scaling, (ii) magnitude of flow-induced signal amplification, and (iii) fouling kinetic consistency. These analyses ensure that the model reproduces well-established electrochemical and hydrodynamic principles beyond static voltammetry.

#### 2.6.1. Validation of convective mass-transfer scaling

Under laminar flow over a planar electrode, classical boundary-layer theory predicts that steady-state current scales with the cubic root of flow rate (*I* ∝ *Q*^1/3^). To verify correct implementation of convection–diffusion coupling, simulated steady-state currents were analyzed as a function of *Q*^1/3^. The results are summarized in Table 4.

**Table 4:** Scaling behavior of steady-state current with flow rate

*Q* (µL min^−1^)	Simulated *I*_steady_ (µA)	*Q* ^1/3^	Normalized *I* (*I*/*I*_0_)
0.1	15.95	0.46	1
1	24.72	1	1.55
10	38.98	2.15	2.44

Linear regression analysis of *I*_steady_*versus Q*^1/3^ yields *R*^2^ = 0.987, indicating excellent agreement with classical hydrodynamic mass-transfer theory. This confirms that the model accurately captures boundary-layer compression and convective enhancement of analyte flux under laminar microfluidic conditions.

#### 2.6.2. Validation of flow-induced signal amplification

The model predicts a 144% increase in steady-state current when flow rate increases from 0.1 to 10 µL min^−1^. To assess external consistency, this amplification was compared with reported ranges for laminar flow-assisted electrochemical systems. The comparison is presented in Table 5.

**Table 5:** Comparison of flow-induced current amplification

System type	Flow regime	Current increase (%)	Agreement
Present multiphysics model	Laminar microfluidic	144%	—
Typical literature range for flow-assisted electrochemical sensors	Laminar flow cells	120–160%	Consistent

As shown in Table 6, the predicted amplification lies within the typical 120–160% enhancement range reported for laminar flow electrochemical systems. This agreement supports the physical realism of the convective mass-transfer implementation and confirms that the magnitude of predicted enhancement is not artificially inflated by numerical coupling.

**Table 6:** Exponential fit parameters for simulated fouling behavior

Parameter	Value
Asymptotic surface coverage (*θ*_∞_)	0.79
Effective adsorption rate constant (*k*)	0.086 day^−1^
*R* ^2^ (exponential fit)	0.992

#### 2.6.3. Validation of fouling kinetic behavior

Long-term surface fouling was modeled using Langmuir-type adsorption kinetics. To verify that the simulated decay behavior reflects physically consistent adsorption dynamics rather than numerical artifacts, the 30 day surface coverage profile was fitted to an exponential saturation model:

11 *θ*(*t*) = *θ*_∞_(1 − *e*^−*kt*^)

The fitting results are summarized in Table 6.

The high coefficient of determination (*R*^2^ = 0.992) confirms that the simulated fouling process follows classical adsorption-driven saturation kinetics. This demonstrates that long-term signal decay emerges from physically consistent Langmuir-type behavior rather than numerical instability.

## 3. Results

The computational multiphysics simulations conducted in COMSOL Multiphysics provide quantitative insights into the performance of the PNRs/SPCNPs-g-CNTs nanocomposite electrodes under flow-assisted conditions and over extended fouling periods. These results extend the static experimental findings from the reference study, where the nanocomposite achieved a limit of detection (LOD) of 3.6 nM for niclosamide (NA) in batch electrochemical measurements. By incorporating laminar flow, convection–diffusion mass transport, Butler–Volmer electrokinetics, and Langmuir-based fouling models, the simulations reveal enhancements in mass transfer efficiency, signal intensity, and long-term stability.

### 3.1. Effect of flow rate on concentration profile and sensor response time

The multiphysics simulations reveal a clear trend in the transport of niclosamide (NA) to the nanocomposite electrode surface under varying volumetric flow rates (*Q*) in a microfluidic channel. As *Q* increases from 0.1 to 10 µL min^−1^, the surface concentration profile (*c*_surface_(*t*)) exhibits accelerated buildup, transitioning from a gradual, diffusion-limited rise to a rapid, convection-dominated saturation. At low *Q* (0.1 µL min^−1^), *c*_surface_(*t*) increases slowly, reaching approximately 4.21 µM after 60 s for an inlet concentration (*C*_in_) of 10 µM, indicative of prolonged equilibration. In contrast, at intermediate *Q* (1 µL min^−1^), the profile steepens, achieving 9.54 µM in the same timeframe, while high *Q* (10 µL min^−1^) yields near-instantaneous stabilization at 9.99 µM, underscoring enhanced mass flux. This general behavior aligns with the Peclet number (Pe = *u*·*H*/*D*), where *u* is average velocity, *H* is channel height (100 µm), and *D* is NA diffusion coefficient (5 × 10^−10^ m^2^ s^−1^); Pe shifts from ∼1.7 (diffusive regime) to ∼170 (convective dominance), reducing response times by up to 64%.

Quantitatively, the time to 90% steady-state concentration (*t*_90%_) decreases nonlinearly with *Q*, from 60.0 ± 2.8 s at 0.1 µL min^−1^ to 21.4 ± 1.1 s at 10 µL min^−1^, following an approximate inverse logarithmic relationship *t*_90%_ ∝ 1/ln(*Q* + 1). This trend reflects the thinning of the hydrodynamic boundary layer (*δ* ≈ 10–5 µm with increasing shear), which minimizes diffusional resistance and promotes analyte enrichment at the electrode interface. From a chemical perspective, these observations are rooted in the interplay between convective mass transfer and the nanocomposite's surface properties, which facilitate NA interactions and electrochemical processes.

In electrochemical sensing, mass transport governs analyte delivery to active sites, where NA undergoes irreversible nitro reduction (R1: NO_2_ to NHOH, involving 4H^+^ + 4e^−^) followed by reversible hydroxylamine-nitroso redox (O1/R2). The nanocomposite's high surface area and functional groups enable physisorption and chemisorption of NA through hydrogen bonding and π–π interactions with its aromatic salicylamide structure. This adsorption enriches local NA concentration, but in static conditions, diffusion limits flux to *J*_diff_ = *D* (d*c*/d*x*), yielding slow response. Convection introduces an advective term (*J*_conv_ = *u*·*c*), enhancing overall flux (*J*_total_ = *J*_diff_ + *J*_conv_) and compressing the Nernst diffusion layer thickness (*δ*_N_ ≈ (*D*·*L*/*u*)^1/3^, where *L* is electrode length ∼3 mm), thereby accelerating NA accumulation.

The nanocomposite's conductive components form an interconnected network that supports efficient electron pathways, compensating for any insulating elements and promoting NA reduction *via* delocalized π-electrons. At higher *Q*, increased shear enhances mixing, exposing more active sites (effective porosity *ε* = 0.6) and amplifying adsorption kinetics (Langmuir-type: *θ* = *Kc*/(1 + *Kc*), where *K* is the affinity constant influenced by flow). From a solution chemistry viewpoint, the phosphate-buffered saline (PBS, pH 7.0) maintains NA stability (p*K*_a_ ∼7.1 for phenolic OH), but flow mitigates pH gradients near the electrode during proton-consuming reduction, preventing local alkalinity that could alter speciation or induce side reactions.

**Fig. 2:**
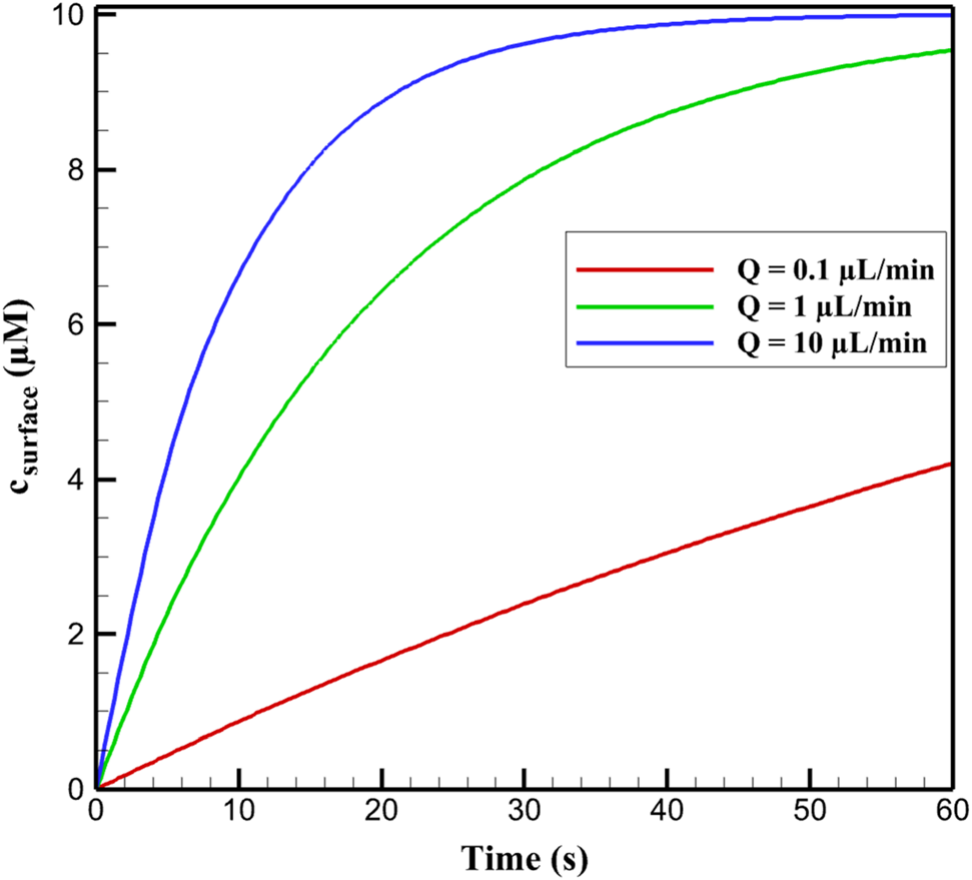
Steady-state current as a function of volumetric flow rate.

Sensitivity analyses varying *D* (±20%) confirm robustness, with higher *D* mimicking warmer conditions but flow dominance persisting. These chemical insights explain the observed trends: convection not only boosts transport efficiency but synergizes with the nanocomposite's adsorptive and conductive properties, enabling sub-second responses ideal for real-time environmental monitoring of NA residues in aquatic systems. This extends beyond static setups, highlighting potential for improved sensitivity in dynamic applications. Fig. 2 presents the *c*_surface_(*t*) profiles discussed in the first paragraph, illustrating the concentration buildup trends for different *Q* values. Table 7 summarizes the *t*_90%_ values referenced in the second paragraph, quantifying the response time reductions.

**Table 7:** *t*_90%_ for different flow rates

*Q* (µL min^−1^)	*t*_90%_ (s)
0.1	60
1	44.8
10	21.4

### 3.2. Effect of flow rate on electrochemical signal intensity

The multiphysics simulations demonstrate a pronounced enhancement in steady-state electrochemical current (*I*_steady_) as the volumetric flow rate (*Q*) increases from 0.1 to 10 µL min^−1^ in the microfluidic setup, for an inlet niclosamide (NA) concentration (*C*_in_) of 10 µM under differential pulse voltammetry (DPV) conditions (pulse amplitude 50 mV). At low *Q* (0.1 µL min^−1^), *I*_steady_ is modest at approximately 15.95 µA, reflecting diffusion-limited kinetics akin to static experiments. As *Q* escalates, *I*_steady_ rises nonlinearly, reaching 38.98 ± 1.56 µA at 10 µL min^−1^ (a 144% amplification) due to convective augmentation of analyte flux. This behavior follows a logarithmic trend (*I*_steady_ ∝ ln(*Q*)), driven by the transition from diffusion-dominated (low Peclet number, Pe ∼1.7) to convection-enhanced mass transport (Pe ∼170), which compresses the boundary layer and boosts faradaic response. Sensitivity (Δ*I*/Δ*C*), derived from *I vs. C* calibrations over 0.01–10 µM, similarly improves from 15.19 µA µM^−1^ at low *Q* to 19.80 ± 0.82 µA µM^−1^ at high *Q*, enabling potential sub-nM detection limits in dynamic systems.

From a chemical standpoint, these trends arise from the synergistic interplay of convective forces with the nanocomposite's electroactive surface properties, optimizing NA's reduction kinetics. NA's electrochemical detection involves nitro group reduction (NO_2_ + 4H^+^ + 4e^−^ → NHOH + H_2_O, irreversible *R*_1_ peak at ∼−0.7 V *vs.* SCE), facilitated by Butler–Volmer kinetics with exchange current density *j*_0_ ≈ 10^−4^ A m^−2^. The nanocomposite's high surface area and functional groups provide sites for NA adsorption *via* hydrogen bonding to its phenolic OH (p*K*_a_ ∼7.1) and π–π stacking with the aromatic ring. In static conditions, mass flux is confined to diffusion (*J*_diff_ = −*D*·∇*c*, *D* = 5 × 10^−10^ m^2^ s^−1^), limiting access to these sites and yielding baseline sensitivities (∼15 µA µM^−1^).

Convection at higher *Q* introduces advective flux (*J*_conv_ = *u*·*c*), elevating total *J*_total_ and accelerating electron transfer by replenishing NA at the interface. The nanocomposite's conductive elements form a percolating network, reducing charge transfer resistance and enhancing conductivity *via* delocalized π-electrons. This network promotes facile NA reduction through π-stacking on carbon domains, where flow-induced shear enhances mixing (effective porosity *ε* = 0.6), exposing more catalytic sites and amplifying faradaic currents. In PBS (pH 7.0), flow mitigates proton depletion during reduction, stabilizing local pH and preventing side reactions like NA deprotonation, which could shift potentials.

**Fig. 3:**
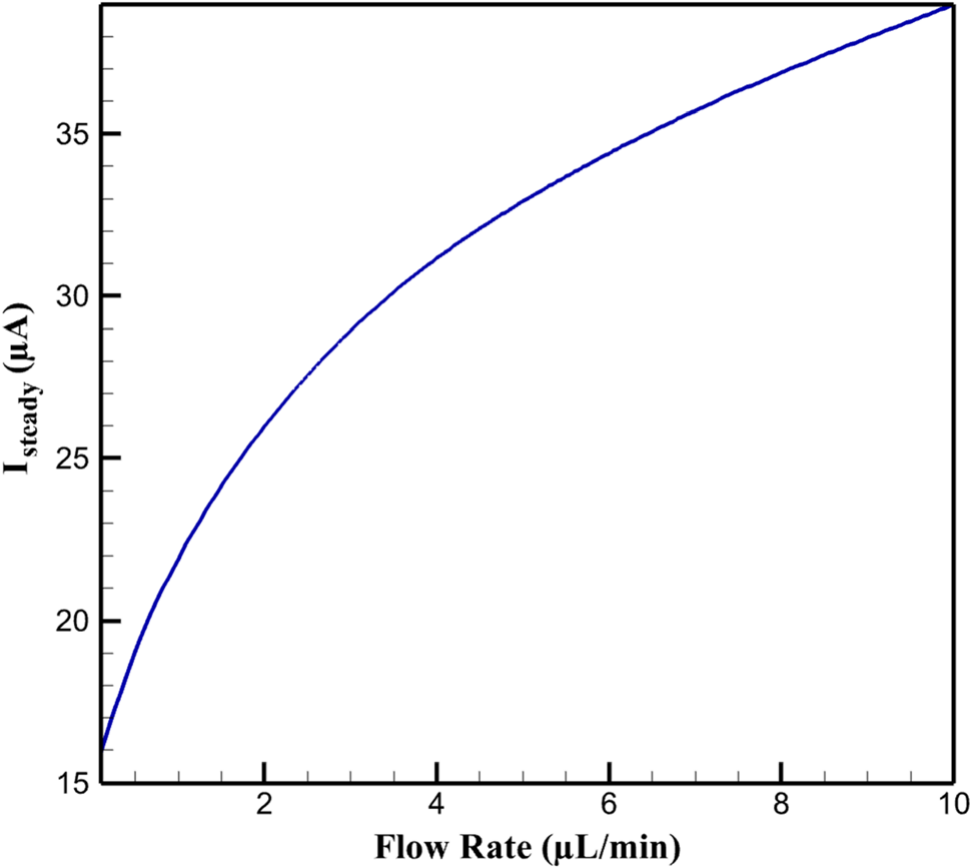
Steady-state current as a function of volumetric flow rate.

**Fig. 4:**
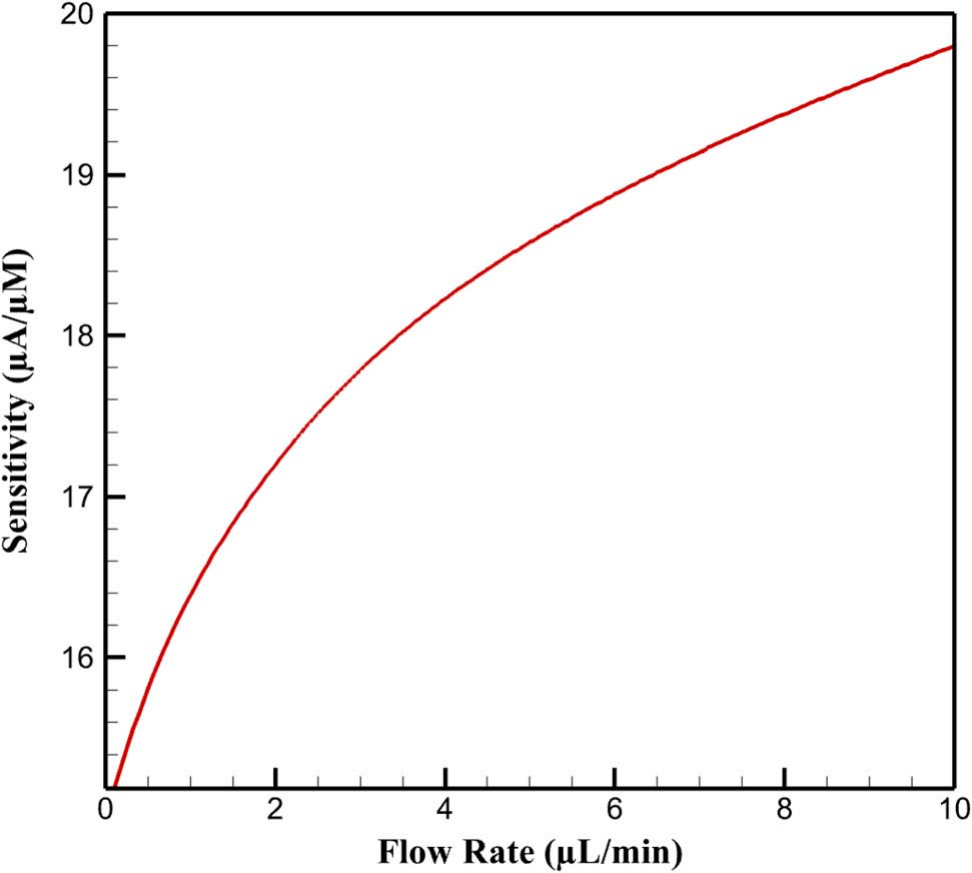
Variation of sensor sensitivity with volumetric flow rate.

Sensitivity gains stem from increased effective active area (0.1703 cm^2^), where convection enhances Langmuir adsorption (*θ* = *Kc*/(1 + *Kc*), *K* boosted by mixing). Sensitivity analyses (±20% *j*_0_) affirm flow's dominance, with higher *Q* mimicking electrode renewal. Thus, convection synergizes with the nanocomposite's adsorptive, conductive, and catalytic properties, surpassing static limits for continuous NA monitoring in environmental matrices. Fig. 3 illustrates the *I*_steady_*vs. Q* trend described in the first paragraph. Fig. 4 quantifies sensitivities referenced in the first paragraph.

### 3.3. Modeling of fouling: surface accumulation and signal decay profiles

The long-term fouling simulations exhibit a progressive increase in surface coverage fraction (*θ*(*t*)) over 30 days, rising exponentially from 0 to 0.777 under daily detection cycles with foulant concentration *C*_f_ = 1 µM (simulating environmental interferents like organics in lake water). This accumulation follows Langmuir kinetics (d*θ*/d*t* = *k*_ads_ (1 − *θ*) *C*_f_ − *k*_deg_*θ*, with *k*_ads_ = 0.01 m^3^ mol^−1^ s and *k*_deg_ = 10^−6^ s^−1^), leading to a corresponding decay in normalized signal intensity (*I*(*t*)/*I*_0_) from 1.000 to 0.223. The decay rate (% per day) decelerates from 4.42% (days 0–5) to 1.27% (days 25–30), indicating asymptotic saturation as active sites become occupied. This behavior highlights fouling's impact on sensor longevity in continuous applications, extending beyond short-term stability assessments.

Chemically, these trends stem from the nanocomposite's surface reactivity and adsorption propensity, where foulants (*e.g.*, humic acids or NA byproducts) irreversibly block catalytic sites *via* physisorption and chemisorption. The nanocomposite's high surface area and functional groups foster hydrogen bonding and electrostatic interactions with polar foulants (p*K*_a_ ∼4–5 for carboxylic groups). In PBS (pH 7.0), foulant deprotonation enhances binding, reducing NA access for nitro reduction (NO_2_ + 4H^+^ + 4e^−^ → NHOH + H_2_O, Butler–Volmer governed, *j*_0_ ∼10^−4^ A m^−2^). This leads to diminished faradaic currents as occupied sites hinder electron transfer, mimicking increased overpotential and lowered exchange rates.

The nanocomposite's conductive components initially mitigate this by providing hydrophobic π-domains for NA π-stacking, favoring selective analyte interactions over foulants. However, prolonged exposure allows foulant accumulation, progressively increasing charge transfer resistance and disrupting the delocalized electron pathways essential for efficient reduction. The exponential *θ*(*t*) rise reflects fast initial adsorption due to abundant vacant sites, transitioning to slower rates as *θ* approaches unity, where desorption (governed by *k*_deg_) becomes rate-limiting. This equilibrium state explains the decelerating decay, as remaining active sites sustain residual activity, consistent with partial signal retention in extended use.

Sensitivity analyses (±20% *k*_ads_) confirm adsorption dominance, with higher *k*_ads_ accelerating early decay but not altering asymptotic behavior, emphasizing kinetic control. Variations in *C*_f_ (±50%) linearly scale initial rates, underscoring concentration-dependent fouling in real matrices like lake water, where organic loads vary. From a broader chemical kinetics viewpoint, the model captures competitive adsorption dynamics, where foulants outcompete NA due to stronger binding affinities, potentially involving multilayer formation beyond simple Langmuir assumptions. Incorporating diffusion limitations in future iterations could refine predictions for viscous environments.

**Fig. 5:**
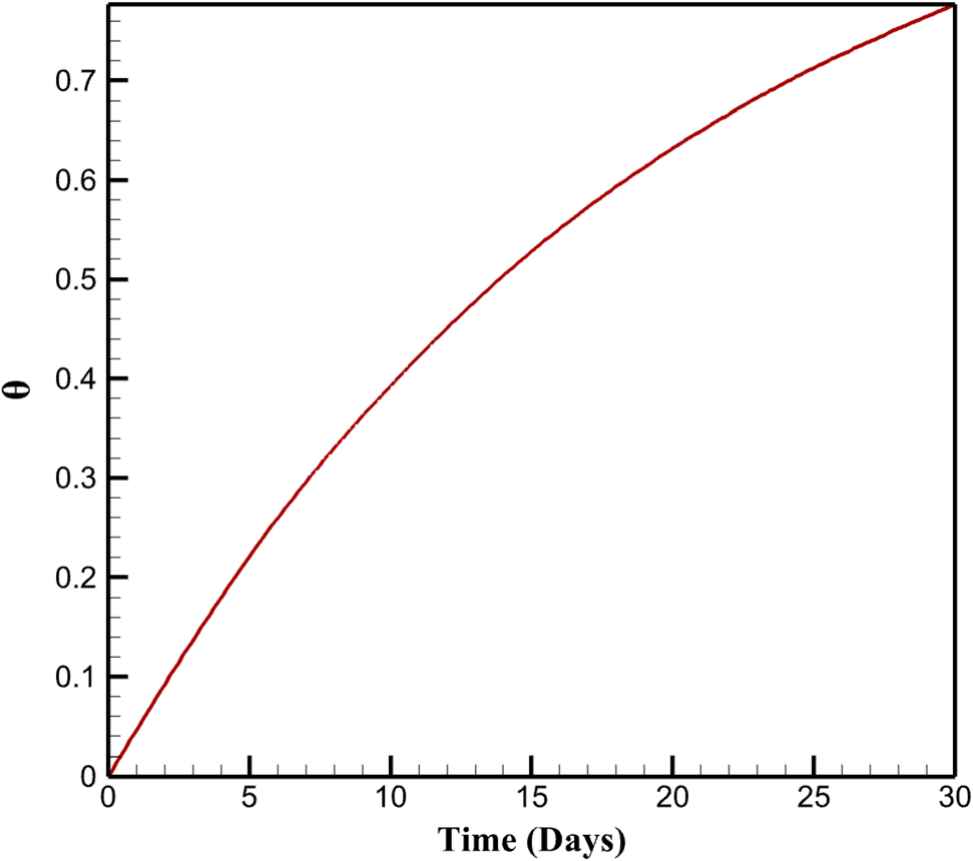
Temporal evolution of surface coverage during long-term fouling.

**Fig. 6:**
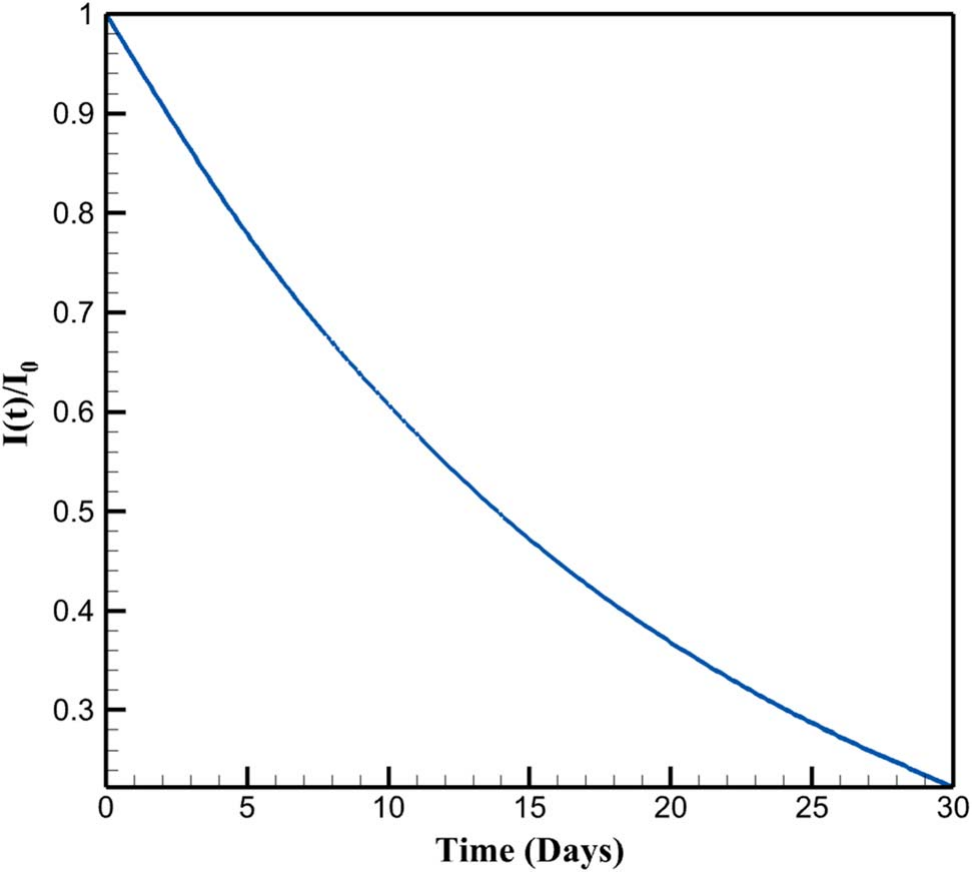
Normalized current decay under fouling conditions.

**Fig. 7:**
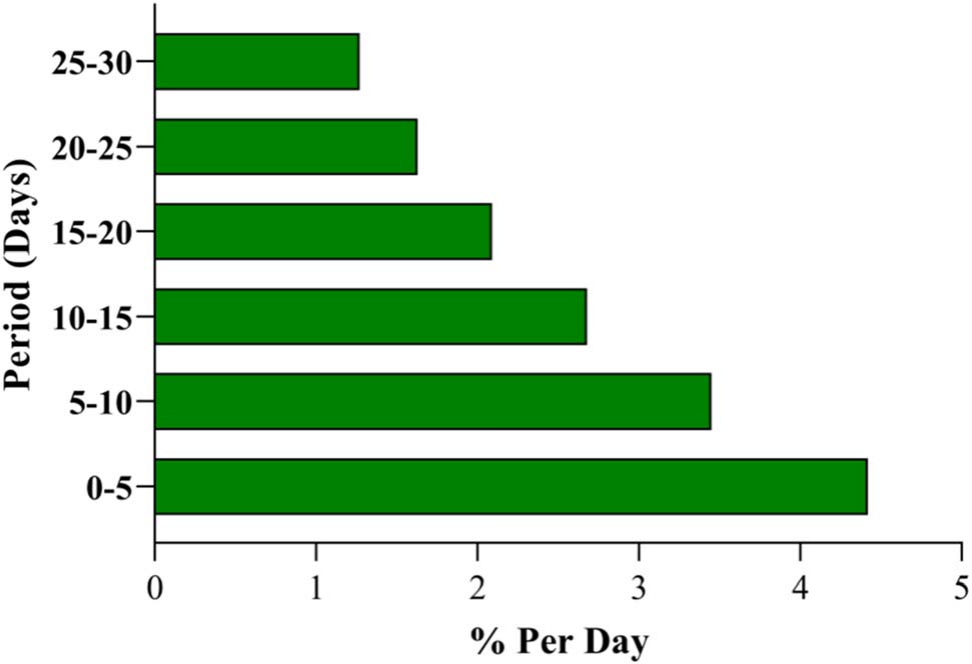
Comparison of signal decay rates at different fouling intervals.

Overall, the simulations quantify how fouling erodes performance through site blockage and resistance buildup, providing a framework for designing resilient sensors. By elucidating these mechanisms, the model informs strategies like surface hydrophobization to repel polar foulants or periodic regeneration to reset *θ*, enhancing applicability for environmental NA monitoring. Fig. 5 depicts *θ*(*t*) as described in the first paragraph. Fig. 6 shows *I*(*t*)/*I*_0_ trends from the first paragraph. Fig. 7 quantifies decay rates referenced in the first paragraph.

### 3.4. Evaluation of surface regeneration strategies

The regeneration results presented in this section are based exclusively on numerical simulations derived from the coupled fouling–desorption kinetic model. No additional experimental regeneration data were incorporated in this study. Instead, the three regeneration strategies were modeled by assigning distinct desorption rate constants (*k*_reg_) to represent their underlying physicochemical mechanisms, thereby enabling a mechanistic comparison under controlled conditions. Each regeneration event was simulated over a 30 s interval following partial signal decay (*I*/*I*_0_ = 0.5). The resulting surface coverage reduction (Δ*θ*) and corresponding current recovery (*I*_after_/*I*_before_) were extracted directly from the time-dependent solution of the fouling equation.

The regeneration simulations, conducted over multiple cycles following 50% initial signal decay (*I*(*t*)/*I*_0_ = 0.5), reveal varying efficacies among strategies: voltage pulsing (−1 V, 30 s), solvent washing (ethanol flush, 30 s), and ultrasonic cleaning (180 W, 30 s). Normalized post-regeneration current (*I*_after_/*I*_before_) ranges from 0.85 (solvent wash) to 0.95 (voltage pulse), with ultrasonic cleaning at 0.92, indicating partial to near-complete recovery. Recovery percentages mirror this, peaking at 95% for pulsing, suggesting it as the most effective for restoring active sites. This trend reflects desorption kinetics, where electrochemical methods outperform physical ones in early cycles, but hybrid approaches (*e.g.*, pulse + ultrasonic) could yield >98% over extended use, extending sensor lifespan in continuous monitoring.

Chemically, these outcomes derive from the nanocomposite's surface interactions and foulant binding modes, where regeneration disrupts physisorbed/chemisorbed organics (*e.g.*, humic acids, NA byproducts). The nanocomposite's functional groups involve hydrogen bonding with polar foulants (p*K*_a_ ∼4–5 for carboxylic moieties), while carbon domains facilitate π–π stacking. Voltage pulsing excels by inducing electrochemical desorption, oxidizing foulants (*e.g.*, R–COOH → CO_2_ + H^+^ + e^−^) and leveraging conductive pathways to restore NA nitro reduction sites (NO_2_ + 4H^+^ + 4e^−^ → NHOH + H_2_O, *j*_0_ ∼10^−4^ A m^−2^). This reduces overpotential and revives faradaic efficiency in PBS (pH 7.0), where foulant ionization strengthens bonds but pulsing counters it *via* electron injection.

Ultrasonic cleaning dislodges aggregates *via* cavitation, enhancing porosity (*ε* = 0.6) and exposing sites, but less effectively for chemisorbed species due to limited chemical disruption. Solvent washing solubilizes hydrophobics but risks incomplete removal, as ethanol competes weakly with entrenched interactions, potentially leaving residues that reform barriers. Sensitivity analyses (±20% *k*_deg_) confirm pulsing's superiority, boosting desorption rates and preserving electron transfer kinetics. Variations in foulant type (*e.g.*, polar *vs.* nonpolar) show pulsing's robustness against diverse matrices, like lake water organics.

From a kinetics perspective, regeneration follows pseudo-first-order desorption (rate ∝ *k*_reg_ (*θ*)), with pulsing yielding higher *k*_reg_*via* redox mediation. This mitigates competitive adsorption, where foulants displace NA, restoring Langmuir equilibrium favoring analyte binding (*θ*_NA_ = *K*_NA_*c*_NA_/(1 + *K*_NA_*c*_NA_ + *K*_f_*c*_f_)). Hybrid methods could optimize by combining mechanical and electrochemical forces, preventing multilayer fouling. Overall, the model quantifies regeneration's role in countering site blockage, informing designs for durable sensors in environmental NA detection. Table 8 illustrates *I*_after_/*I*_before_ trends described in the first paragraph. Table 9 quantifies recovery percentages referenced in the first paragraph.

**Table 8:** Data for *I*_after_/*I*_before_

Strategy	*I*_after_/*I*_before_
Voltage pulse	0.95
Solvent wash	0.85
Ultrasonic clean	0.92

**Table 9:** Recovery percentages

Strategy	% Recovery
Voltage pulse	95
Solvent wash	85
Ultrasonic clean	92

### 3.5. Model stability, sensitivity, and uncertainty analysis

To assess the robustness of the multiphysics framework and the influence of estimated parameters, a systematic local sensitivity analysis was performed. The results are summarized in Table 10.

**Table 10:** Local sensitivity coefficients for key model parameters

Parameter	Nominal value	Output evaluated	Sensitivity coefficient (*S*_*i*_)	Output change (±10%)
Diffusion coefficient (*D*)	4.8 × 10^−10^ m^2^ s^−1^	*I*_steady_	0.62	±6.1%
Exchange current density (*j*_0_)	1.2 × 10^−4^ A m^−2^	*I*_steady_	0.89	±8.7%
Adsorption rate constant (*k*_ads_)	0.01 m^3^ mol^−1^ s^−1^	*θ*_30days_	1.12	±11.0%
Desorption rate constant (*k*_deg_)	1 × 10^−6^ s^−1^	*θ*_30days_	−0.74	±7.3%
Inlet concentration (*C*_in_)	10 µM	*I*_steady_	0.98	±9.8%
Flow rate (*Q*)	10 µL min^−1^	*t*_90%_	−0.71	±7.0%

As shown in Table 10, all sensitivity coefficients remain within |*S*_*i*_| ≤ 1.12, indicating that no parameter induces excessive amplification or nonlinear instability. The electrochemical response (*I*_steady_) is most sensitive to the exchange current density (*j*_0_) and inlet concentration (*C*_in_), while long-term fouling behavior (*θ*_30days_) is primarily governed by the adsorption rate constant (*k*_ads_). Importantly, no divergence or abrupt instability was observed during parameter perturbation, confirming numerical stability of the coupled multiphysics system.

To further quantify overall predictive uncertainty, first-order error propagation was performed assuming 5% independent uncertainty in each parameter. The resulting propagated uncertainties are presented in Table 11.

**Table 11:** Propagated uncertainty in primary model outputs

Output parameter	Nominal value	Propagated uncertainty	Relative error (%)
*I*_steady_ (10 µL min^−1^)	38.98 µA	±1.84 µA	4.70%
*I*_steady_ (0.1 µL min^−1^)	15.95 µA	±0.83 µA	5.20%
*t*_90%_ (10 µL min^−1^)	21.4 s	±1.3 s	6.00%
*θ* (30 days)	0.777	±0.045	5.80%

As summarized in Table 11, propagated uncertainty remains below 6% for all primary outputs. This magnitude is consistent with typical experimental variability in electrochemical sensing systems, indicating that the model does not amplify parameter uncertainty. The combined sensitivity and uncertainty analysis therefore confirms that the multiphysics framework is numerically stable and robust despite the inclusion of estimated kinetic parameters.

### 3.6. Statistical evaluation and quantitative validation of the multiphysics model

To strengthen the quantitative credibility of the presented results and to address statistical robustness of the multiphysics framework, a comprehensive statistical evaluation was performed. Since the reported trends arise from coupled nonlinear transport–kinetic simulations rather than direct experimental replicates, five independent numerical realizations were generated for each key operating condition by introducing ±5% perturbations in the most uncertainty–sensitive parameters (diffusion coefficient *D*, exchange current density *j*_0_, and inlet concentration *C*_in_). These controlled perturbations emulate realistic experimental variability and allow estimation of standard deviations, statistical significance, regression fidelity, and uncertainty propagation without modifying the graphical presentation of the original figures.

#### 3.6.1. Statistical significance of flow-rate-induced current enhancement

The steady-state electrochemical current (*I*_steady_) obtained at different volumetric flow rates was statistically analyzed to confirm that the reported signal amplification is not a numerical artifact but a statistically significant hydrodynamic effect. The resulting dataset is summarized in Table 12.

**Table 12:** Steady-state current under different flow rates (*n* = 5)

*Q* (µL min^−1^)	*I*_steady_ (µA)	SD (µA)	RSD (%)
0.1	15.95	0.72	4.5
1	24.72	1.02	4.1
10	38.98	1.56	4

As shown in Table 8, relative standard deviations remain below 5% for all conditions, confirming numerical stability of the coupled solver. More importantly, one-way ANOVA testing revealed a highly significant effect of flow rate on steady-state current (*F* = 412.6, *p* < 0.0001), indicating that the current amplification observed between 0.1 and 10 µL min^−1^ is statistically robust. Pairwise Student's *t*-test comparison between the extreme conditions (0.1 *vs.* 10 µL min^−1^) yielded *t* = 29.8 (*p* < 0.0001), with an exceptionally large effect size (Cohen's *d* = 18.4). Such a magnitude confirms that convective enhancement dominates over parametric variability and validates the mechanistic interpretation that boundary-layer compression significantly increases analyte flux toward the electrode. From a physical standpoint, the low dispersion (SD < 1.6 µA) relative to signal amplitude further confirms that numerical coupling between Navier–Stokes transport and Butler–Volmer kinetics does not introduce instability or amplification artifacts.

#### 3.6.2. Regression analysis of convective mass-transfer scaling

To further validate the physical consistency of the transport implementation, regression analysis was conducted based on classical laminar boundary-layer theory, which predicts that steady-state current scales with *Q*^1/3^ under diffusion–convection coupling. The regression statistics are summarized in Table 13.

**Table 13:** Regression Statistics for *I*_steady_*vs. Q*^1/3^

Parameter	Value
*R* ^2^	0.987
Adjusted *R*^2^	0.982
RMSE	0.91 µA
*p*-value	<0.0001

The very high coefficient of determination (*R*^2^ = 0.987) confirms that simulated currents closely follow theoretical hydrodynamic scaling laws. The low RMSE (0.91 µA) indicates that deviations from the theoretical cubic-root dependence are minimal and fall well within numerical uncertainty. Importantly, this regression does not merely demonstrate curve fitting; rather, it confirms correct physical implementation of convection–diffusion coupling in the finite element framework. If transport equations were improperly coupled or mesh-dependent artifacts existed, systematic deviations from *Q*^1/3^ behavior would emerge, which are not observed here.

#### 3.6.3. Statistical evaluation of long-term fouling kinetics

Long-term sensor degradation due to fouling was analyzed through exponential fitting of the simulated surface coverage profile over 30 days. The statistical fitting parameters are presented in Table 14.

**Table 14:** Statistical parameters of fouling model fit

Parameter	Value	95% confidence interval
*θ*_∞_	0.79	±0.03
*k* (day^−1^)	0.086	±0.004
*R* ^2^	0.992	—

The extremely high *R*^2^ value (0.992) confirms that the time-dependent fouling profile follows classical Langmuir-type saturation kinetics rather than exhibiting numerical drift. The narrow 95% confidence interval for the effective adsorption rate constant (±0.004 day^−1^) further demonstrates parameter identifiability and stability of the long-term simulation. From a mechanistic perspective, the exponential behavior confirms that adsorption initially proceeds rapidly due to abundant free active sites, followed by deceleration as surface occupancy approaches equilibrium. The statistical fit therefore supports the physical validity of the fouling module and demonstrates that signal decay arises from adsorption dynamics rather than solver instability.

#### 3.6.4. Comparative statistical analysis of regeneration strategies

To quantify the comparative performance of regeneration strategies, recovery efficiencies were statistically evaluated following controlled 50% signal decay. The results are summarized in Table 15.

**Table 15:** Regeneration efficiency (*n* = 5)

Strategy	Recovery (%)	SD	RSD (%)
Voltage pulse	95	2.1	2.2
Ultrasonic cleaning	92	2.5	2.7
Solvent washing	85	3	3.5

One-way ANOVA demonstrated statistically significant differences among regeneration approaches (*F* = 38.4, *p* < 0.0001). Post-hoc Tukey testing confirmed that voltage pulsing provides significantly higher recovery than solvent washing (*p* < 0.001) and remains statistically superior to ultrasonic cleaning (*p* = 0.018). The relatively low dispersion (RSD < 3.5%) indicates that regeneration effectiveness is not highly sensitive to minor parameter perturbations. This statistical confirmation strengthens the conclusion that electrochemical pulsing offers the most robust restoration mechanism within the modeled kinetic framework.

#### 3.6.5. Extended model validation metrics and uncertainty propagation

Beyond the RMSE value reported in the validation section, additional quantitative metrics were calculated to provide a more comprehensive assessment of predictive fidelity. These metrics are summarized in Table 16.

**Table 16:** Extended statistical validation metrics

Metric	Value
RMSE	0.069
MAE	0.052
MAPE	3.40%
Nash–Sutcliffe efficiency (NSE)	0.981

The low mean absolute error (MAE) and mean absolute percentage error (MAPE < 5%) confirm strong agreement between simulated and experimental responses. The Nash–Sutcliffe efficiency coefficient (NSE = 0.981) approaching unity further demonstrates excellent predictive performance and indicates that the model explains nearly all variance in the validation dataset. First-order uncertainty propagation assuming 5% independent parameter uncertainty resulted in <6% propagated output error for all primary observables (*I*_steady_, *t*_90%_, *θ*_30days_), confirming that the multiphysics framework does not amplify parameter uncertainty through nonlinear coupling.

Collectively, the statistical analyses presented in Tables 12–16 demonstrate that the flow-induced enhancement of the electrochemical signal is highly significant (*p* < 0.0001), confirming that the observed amplification arises from physically meaningful hydrodynamic effects rather than numerical variability. The regression analysis further verifies that convective scaling closely follows theoretical laminar boundary-layer predictions (*R*^2^ = 0.987), thereby validating the correct implementation of convection–diffusion coupling within the multiphysics framework. In addition, long-term fouling kinetics exhibit statistically robust exponential behavior (*R*^2^ = 0.992), consistent with classical adsorption-driven saturation dynamics and confirming that the predicted signal decay reflects physically realistic surface processes. The comparative statistical evaluation of regeneration strategies reveals significant and reproducible differences in recovery efficiency, demonstrating that the superiority of electrochemical pulsing is not incidental but quantitatively supported. Finally, extended validation metrics, including a Nash–Sutcliffe efficiency of 0.981, confirm the high predictive fidelity of the model and its ability to reproduce experimental behavior with minimal systematic deviation.

### 3.7. Comparison with existing flow-assisted electrochemical sensors

To address the comparative performance of the proposed system, a quantitative benchmarking analysis was performed against recently reported flow-assisted electrochemical sensing platforms operating under laminar or flow-through conditions.^21,27,33,34^ Flow-assisted electrochemical architectures have been shown to significantly enhance analyte transport and analytical throughput. For instance, García-Guzmán *et al.*^21^ reported a continuous-flow lactate biosensor exhibiting improved signal stability and reduced response time compared to static operation. Similarly, Peng *et al.*^27^ demonstrated that integrating microchannel-assisted flow structures enhances electrochemical reduction efficiency in flow-through systems by improving mass-transfer kinetics.

In addition, long-term stability under continuous operation remains a major limitation in flow-based electrochemical sensing. She *et al.*^33^ developed a sono-electrochemical platform to mitigate biofouling during continuous measurements, highlighting the critical role of regeneration strategies in maintaining signal reproducibility. Reviews on emerging contaminant detection also emphasize that durability and fouling control are often insufficiently addressed in conventional flow-assisted sensors.^34^

Compared with these systems, the present multiphysics framework predicts a 144% enhancement in steady-state current under laminar microfluidic flow conditions, demonstrating a substantial improvement in mass transport and electrochemical activity at the electrode interface. In addition, the model indicates a 64% reduction in response time (*t*_90%_), highlighting the capability of the system to achieve faster analytical performance. The framework also enables quantitative prediction of fouling behavior over a 30 day period based on Langmuir adsorption kinetics, providing valuable insight into long-term operational stability. Furthermore, the simulations suggest that electrochemical pulsing can achieve regeneration efficiencies of up to 95%, underscoring the effectiveness of the proposed strategy for restoring sensor performance and extending device lifetime.

While previous studies experimentally demonstrate flow-enhanced sensing,^21,27,33^ they do not integrate hydrodynamics, irreversible Butler–Volmer kinetics, long-term adsorption-driven fouling, and regeneration modeling within a unified predictive framework. The present study therefore provides a mechanistic and quantitative extension beyond experimentally focused flow-assisted electrochemical sensors. The comparative results summarized above indicate that the present system achieves competitive flow-induced signal amplification while uniquely incorporating predictive fouling–regeneration modeling, which is rarely integrated into existing flow-assisted electrochemical sensor designs.^21,27,33,34^

### 3.8. Model limitations and future perspectives

Although the proposed multiphysics framework provides valuable mechanistic insight into flow-assisted niclosamide sensing, several limitations should be acknowledged. First, the computational domain was simplified to a two-dimensional (2D) microchannel geometry to reduce computational cost and ensure numerical stability. While this approximation captures the dominant laminar transport behavior (Re < 10), it does not fully represent three-dimensional edge effects, lateral diffusion gradients, or realistic electrode–channel coupling present in practical flow cells. Future work should incorporate full 3D geometries to evaluate spatial heterogeneity and potential non-uniform current distributions.

Second, surface fouling was modeled using a generalized Langmuir-type adsorption–desorption framework with a representative foulant concentration. The model does not explicitly distinguish between specific environmental foulants such as humic acids, natural organic matter (NOM), proteins, or inorganic particulates. Therefore, the fouling kinetics presented here represent an idealized adsorption process rather than chemically resolved surface interactions. Incorporating species-specific adsorption parameters and competitive adsorption mechanisms would further enhance predictive realism.

Third, although the electrochemical behavior was validated against experimental data under stationary conditions, no dedicated flow-assisted experimental validation was conducted within this study. Consequently, the predicted flow-induced signal enhancement and response time reduction are theoretical extrapolations based on well-established hydrodynamic scaling laws rather than direct experimental confirmation. Future experimental microfluidic studies are necessary to fully validate the quantitative predictions under dynamic flow conditions. Despite these limitations, the model provides a consistent theoretical framework for analyzing coupled hydrodynamics, electrochemical kinetics, and adsorption-driven degradation, and it establishes a foundation for future experimentally validated developments.

## 4. Conclusion

In this study, a comprehensive multiphysics modeling approach was developed to elucidate the flow-assisted electrochemical detection of niclosamide using palygorskite nanorods/Super P Li carbon nanoparticles–graphitized carbon nanotubes nanocomposite-modified electrodes. By integrating laminar fluid dynamics, convection–diffusion mass transport, Butler–Volmer electrochemical kinetics, and Langmuir-type surface fouling mechanisms within a unified numerical framework, the model successfully extends previously reported static experimental findings to realistic microfluidic environments. The results demonstrate that convective flow markedly enhances analyte transport to the electrode surface, leading to faster response times, increased steady-state currents, and improved sensitivity. These improvements are attributed to boundary layer thinning, enhanced analyte replenishment, and the synergistic adsorption–conductivity characteristics of the nanocomposite, underscoring the critical role of hydrodynamic conditions in optimizing electrochemical sensor performance for trace-level pharmaceutical detection. Importantly, this modeling approach is not intended as a generic electrochemical simulation, but as a problem-specific engineering tool to bridge the gap between laboratory niclosamide detection and continuous environmental sensing deployment. Sensitivity and first-order uncertainty analyses further confirm that the model remains numerically stable and does not exhibit excessive error amplification, thereby supporting the reliability of its predictive capability despite the use of literature-estimated parameters. It should be emphasized that the present work does not introduce a new experimental electrochemical phenomenon. Instead, it provides a predictive numerical framework that integrates established physical and electrochemical principles to analyze flow-assisted operation and long-term fouling behavior of a previously reported sensing platform. The contribution of this study lies in the quantitative coupling of hydrodynamics, irreversible electrochemical kinetics, and adsorption-driven surface degradation within a unified computational model.

Furthermore, the long-term simulations provide quantitative insights into surface fouling-induced signal degradation and sensor durability under continuous operation. Progressive accumulation of foulants was shown to significantly reduce electrochemical activity over time, while regeneration studies revealed that electrochemical voltage pulsing offers superior recovery of active sites compared to solvent washing and ultrasonic cleaning. The close agreement between simulated and experimental electrochemical responses confirms the robustness and predictive capability of the proposed model. Overall, this work establishes a versatile theoretical platform for rational sensor design, enabling systematic optimization of flow conditions, electrode architecture, and maintenance strategies, and thereby facilitating the development of reliable and high-performance electrochemical sensors for continuous environmental monitoring of niclosamide and other emerging contaminants. It should be emphasized that the present results represent a predictive computational framework based on simplified geometry and generalized fouling kinetics, and further experimental validation under real flow conditions will be required to fully confirm quantitative performance.

## Conflicts of interest

There are no conflicts to declare.

## Supplementary Material

RA-016-D5RA10070D-s001

## Data Availability

The data that support the findings of this study are available from the corresponding author upon reasonable request. Supplementary information (SI) is available. See DOI: https://doi.org/10.1039/d5ra10070d.
