# Supplementary material for: Multiphysics insights into flow-assisted electrochemical sensing of niclosamide: effects of surface fouling and regeneration
Source: RSC Adv. 2026 Mar 13;16(15):13785–800. doi: 10.1039/d5ra10070d (PMC12983465; doi:10.1039/d5ra10070d)
Supplement: RA-016-D5RA10070D-s001 [file RA-016-D5RA10070D-s001.pdf]

This journal is © The Royal Society of Chemistry 2026

Supplementary Information

S1. Experimental Basis of Model Validation (Extracted from [39])

This study employs the experimental electrochemical platform reported in [39] as the validation benchmark for the numerical model. For transparency and reproducibility, the essential experimental details are summarized below.

S2. Materials and Reagents

Niclosamide (analytical grade) was used without further purification.

Palygorskite nanorods (PNRs), Super P carbon nanoparticles (SPCNPs), and graphitized carbon nanotubes (g-CNTs) were employed for fabrication of the nanocomposite sensing layer.

Phosphate buffer saline (PBS, 0.1 M, pH 7.0) was prepared using analytical-grade reagents and deionized water.

All solutions were prepared freshly prior to electrochemical measurements.

S3. Fabrication of the PNRs/SPCNPs–g-CNTs Modified Electrode

The nanocomposite was prepared by dispersing appropriate ratios of PNRs, SPCNPs, and g-CNTs in ethanol followed by ultrasonication to obtain a homogeneous suspension.

A glassy carbon electrode (GCE) with a diameter of 3 mm was polished sequentially using alumina slurry, rinsed thoroughly with deionized water, and dried at room temperature.

A defined volume of the nanocomposite suspension was drop-cast onto the polished GCE surface and allowed to dry at ambient conditions, forming the PNRs/SPCNPs–g-CNTs/GCE working electrode.

S4. Electrochemical Measurements

Electrochemical experiments were conducted using a conventional three-electrode configuration:

- Working electrode: PNRs/SPCNPs–g-CNTs/GCE
- Reference electrode: Ag/AgCl
- Counter electrode: Platinum wire

Measurements were performed using Differential Pulse Voltammetry (DPV) in 0.1 M PBS (pH 7.0) at 25 °C.

Operational parameters included:

- Pulse amplitude: 50 mV

- Diffusion-controlled measurement conditions
- Ambient laboratory temperature

S5. Electrochemical Characteristics Relevant to Modeling

The following experimentally determined parameters were used as boundary conditions for numerical simulations:

**Parameter**	**Value**
Electrode diameter	3 mm
Geometric area	0.0707 cm^2
Electroactive area	0.1703 cm^2
Supporting electrolyte	0.1 M PBS (pH 7.0)
Temperature	25 °C (298 K)
Technique	DPV
Pulse amplitude	50 mV
Linear detection range	0.01–10 µM
Limit of detection (LOD)	3.6 nM

The electroactive surface area was calculated experimentally in [39] using the Randles–Ševčík equation based on cyclic voltammetry data.

S6. Model-Related Parameter Determination

To enable numerical simulation of the electrochemical response:

- The exchange current density (j_0) was obtained by fitting simulated peak currents to the experimental calibration curve reported in [39]. The optimized value (1.2×10^{-4} A m^{-2}) reproduced the experimental sensitivity with <5% deviation.
- The charge transfer coefficient was set to $\alpha = 0.5$, assuming an irreversible electrochemical process.
- The diffusion coefficient of niclosamide in PBS was taken as 4.8×10^{-10} m^2 s^{-1}, consistent with reported values for aromatic pharmaceutical compounds in aqueous systems.

These parameters ensure that the numerical model remains physically consistent while fully anchored to the experimental dataset reported in [39].
